# Mangosteen Metabolites as Promising Alpha-Amylase Inhibitor Candidates: In Silico and In Vitro Evaluations

**DOI:** 10.3390/metabo12121229

**Published:** 2022-12-07

**Authors:** Abdelsattar M. Omar, Dana F. AlKharboush, Khadijah A. Mohammad, Gamal A. Mohamed, Hossam M. Abdallah, Sabrin R. M. Ibrahim

**Affiliations:** 1 Department of Pharmaceutical Chemistry, Faculty of Pharmacy, King Abdulaziz University, Jeddah 21589, Saudi Arabia; 2 Department of Pharmaceutical Chemistry, Faculty of Pharmacy, Al-Azhar University, Cairo 11884, Egypt; 3 Center for Artificial Intelligence in Precision Medicines, King Abdulaziz University, Jeddah 21589, Saudi Arabia; 4 Department of Natural Products and Alternative Medicine, Faculty of Pharmacy, King Abdulaziz University, Jeddah 21589, Saudi Arabia; 5 Department of Pharmacognosy, Faculty of Pharmacy, Cairo University, Cairo 11562, Egypt; 6 Department of Chemistry, Preparatory Year Program, Batterjee Medical College, Jeddah 21442, Saudi Arabia; 7 Department of Pharmacognosy, Faculty of Pharmacy, Assiut University, Assiut 71526, Egypt

**Keywords:** diabetes, alpha-amylase, *Garcinia mangostana*, xanthones, industrial development, molecular docking, molecular dynamics

## Abstract

Diabetes is a chronic metabolic disorder characterized by raised glucose levels in the blood, resulting in grave damage over time to various body organs, including the nerves, heart, kidneys, eyes, and blood vessels. One of its therapeutic treatment approaches involves the inhibition of enzymes accountable for carbohydrate digestion and absorption. The present work is aimed at evaluating the potential of some reported metabolites from *Garcinia mangostana* (mangosteen, Guttiferae) as alpha-amylase inhibitors. Forty compounds were assessed for their capacity to inhibit alpha-amylase using in silico studies as well as in vitro assays. Molecular docking was carried out to analyze their binding capacities in the 3D structure of alpha-amylase (PDB ID: 4GQR). Among the tested compounds, 6-O-β-D-glucopyranosyl-2,4,6,3′,4′,6′-hexahydroxybenzophenone (**8**), aromadendrin-8-C-glucoside (**5**), epicatechin (**6**), rhodanthenone (**4**), and garcixanthone D (**40**) had a high XP G.score and a Glide G.score of −12.425, −11.855, −11.135, and −11.048 Kcal/mol, respectively. Compound **8** possessed the XP and Glide docking score of −12.425 Kcal/mol compared to the reference compounds myricetin and acarbose which had an XP and Glide docking score of −12.319 and 11.201 Kcal/mol, respectively. It interacted through hydrogen bond formations between its hydroxyl groups and the residues His 101, Asp 197, Glu 233, Asp 300, and His 305, in addition to water bridges and hydrophobic interactions. Molecular mechanics-generalized born surface area (MM-GBSA) was used to calculate the binding free energy and molecular dynamic studies that indicated the stability of the alpha-amylase-compound **8** complex during the 100 ns simulation in comparison with myricetin- and acarbose-alpha-amylase complexes. Additionally, the in vitro alpha-amylase inhibition assay findings validated the in silico study’s findings. This could further validate the potential of *G. mangostana* as a candidate for diabetes management.

## 1. Introduction

Diabetes mellitus is a metabolic disorder that results from impaired pancreatic β-cells and is characterized by high blood glucose [1,2]. According to some investigations, almost 7 million people are diabetic, and about 3 million are pre-diabetic [3]. The WHO (World Health Organization) has estimated that Saudi Arabia is one of the top three countries in the Middle East and one of the top ten in the world for diabetes rate [4].

It is well-known that diabetes raises the possibility for the development of various critical life-menacing health issues as a consequence of the impairment and disruption in several organs’ functions (hearts, kidneys, skin, nerves, or blood vessels), resulting in micro- and macro-vascular elaborations that involve diabetic retinopathy, nephropathy, and neuropathy, as well as hypertension, atherosclerosis, and strokes [5]. These complications are accountable for the mortality of the majority of diabetic patients. Additionally, elevated levels of blood glucose have been shown to stimulate cancer cell progression and proliferation [6].

Despite the available treatment options, diabetes remains one of the largest health concerns, with an increasing prevalence in recent decades [7]. Several oral hypoglycemic medications are used to manage high blood glucose, including biguanides, sulfonylureas, and acarbose [8]. Acarbose is an FDA-approved drug used for treating type 2 diabetes; it works by inhibiting the HPA (human pancreatic alpha-amylase) and intestinal alpha-glucoside hydrolase competitively [9]. By inhibiting both enzymes, acarbose slows glucose absorption, which results in decreased blood glucose levels [10]. Alpha-amylase functions by catalyzing the α-(1–4) bond hydrolysis in starch and belongs to the glycosyl-hydrolase family-13 [11,12]. Humans have two distinct forms of alpha-amylase that are secreted by the pancreas and salivary glands [13]. These two isoforms have prime functions in digesting starch. Digesting starch starts in the mouth with the HSA (human salivary α-amylase) [14]. Following this step, pancreatic alpha-amylase further digests starch into a mixture of maltotriose, maltose, and other branched small, oligo-saccharides, which are then broken down by α-glucosidases to glucose. For this reason, understanding how the enzyme alpha-amylase works and inhibiting it could be a promising approach for decreasing blood glucose and designing novel inhibitors that specifically prohibit HPA.

The proposed mechanism of alpha-amylase involves several amino acids known as catalytic amino acids. These are two aspartate residues and glutamic acid [15]. According to previous studies, five major binding subsites span the active site of alpha-amylase, as well as one minor subsite [16]. As mentioned above, this enzyme is, in fact, a potential target that has been utilized for treating diabetes.

It has been stated that the usage of the available α-amylase inhibitors (AAIs) is accompanied by undesired adverse effects, including flatulence, abdominal pain, diarrhea, and meteorism [17]. Therefore, research efforts have focused on exploring safe and effective natural AAIs.

Recently, advanced computational investigations have permitted the screening of diverse metabolites to select the potential hits that can be further considered for in vitro verification. In addition, they play a fundamental role in drug development and discovery because of their economic, fast throughput, and labor-saving features compared to their in vivo and in vitro counterparts [18]. Molecular docking is a recognized in silico structure-dependent method that permits the verification of novel agents of therapeutic benefit, the prediction of target–ligand interaction at the molecular level, or the delineation of structure–activity relations [19].

Additionally, it was stated that in vitro estimation has various merits, including the strict monitoring of physical and chemical circumstances, minimized cost, high throughput, and reduced animal usage [20].

Natural metabolites reported from plants, microorganisms, and animals have become more valuable targets for discovering treatments for various health disorders, including diabetes. It is worth noting that natural products are utilized worldwide to manage blood glucose levels in diabetic patients. For example, the extracts of *Cecropia obtusifolia*, *Equisetum myriochaetum*, *Leptolobium panamense*, *Agarista mexicana*, *Cucurbita ficifolia*, *Brickellia veronicaefolia*, *Bauhinia forficate*, *Senna auriculata*, *Abelmoschus esculentus*, and *Parmentiera aculeata* are used in treating diabetes in various countries [21]. Their mechanisms of action are through α-amylase/α-glucosidase prohibition, the modulation of glucose transporters expression and glucose uptake, the stimulation of pancreatic β-cell proliferation and insulin secretion, insulin resistance control, and oxidative stress regulation [22].

Additionally, unique phytoconstituents, such as ginsenosides, berberine, curcumin, gingerols, stevioside, capsaicin, resveratrol, catechins, anthocyanins, genistein, hesperidin, and phenolic compounds, separated from various species revealed anti-diabetes capacities through different mechanisms of action [23].

*Garcinia mangostana* contains diverse classes of metabolites including xanthones, flavonoids, and benzophenones, with an array of bioactivities including antimicrobial, cytotoxic, antioxidant, and antidiabetic activities [24,25,26,27,28,29,30,31,32].

Xanthones are the principal constituents reported from this plant. These metabolites feature a flat, planar tricyclic skeleton of aromatic rings having different functionalities on the A and C rings (e.g., hydroxy, methoxy, or isoprenyl). Xanthones, including oxygenated, isoprenylated glycosides, bisxanthones, and xanthonolignoids, are most associated with the *G. mangostana* fruit [33]. These reported metabolites have been stated to demonstrate diversified bioactivities related to common health disorders. Among them, γ-mangostin and α-mangostin are the major isoprenylated derivatives that are separated from arils, stems, pericarp, and seeds and displayed numerous bioactivities. α-mangostin has been included in different formulations such as supplements, capsules, lotions, and creams that are popular for consumption for various health-promoting purposes [34]. Many studies reported the potential of α-mangostin as a chemo-preventive and anticancer agent. It is evident that α-mangostin acts on specific targets, thereby modulating specific signaling pathways, such as MMPs, CDKs, STAT3, ROS, MAPK, AMPK, JNK2/AP-1/Snail, and PI3K/AKT [34]. Recent in vitro and in silico investigations revealed the potential of α-mangostin to suppress DENV-2 (dengue-virus serotype-2) production at various phases of its replication cycle, and it was thought that it might be used as a therapeutic/prophylactic agent against DENV-2. It was found to interact with different DENV protein targets, including NS2B-NS3 protease, NS5 methyltransferase, and glycoprotein E [35]. Chi et al. proved that α-mangostin and some of its synthesized derivatives revealed significant in vitro potential as AChE (acetylcholinesterase) and BuChE (butyrylcholinesterase) inhibitors [36]. It also induced the improvement of the impaired EDV (endothelium-dependent vasodilation) and up-regulation of the NO/eNOS (nitric oxide/endothelial-nitric-oxide synthase) pathway in diabetic mouse aortas through the inhibition of the aSMase/ceramide pathway, supporting its anti-diabetic capacity [37]. Furthermore, its treatment suppressed aerobic glycolysis in AIA (adjuvant-induced arthritis) rats, resulting in the relief of inflammation-linked hypoxia and the amendment of pathological neovascularization, validating its anti-rheumatic potential [38]. In a docking study, γ-mangostin and α-mangostin revealed potential on aldose reductase, PPAR (peroxisome-proliferator-activated receptor)-γ receptor, and DPP (dipeptidyl-peptidase)-4 enzymes with affinity values similar to the tested ligands [39]. Djeujo et al. demonstrated that α-mangostin possessed a marked α-glucosidase inhibition potential, in addition to its protective action on oxidation damage and protein glycation [38]. In addition to that, α-mangostin nanosponges revealed noticeable α-glucosidase inhibition (IC_50_ 0.9352 µM) which was 3.11-fold bigger than acarbose. In vivo studies showed that α-mangostin-loaded nano-sponges prolonged the plasma antidiabetic response, thus improving patient compliance by the slow release of α-mangostin and less frequent doses needed [40]. γ-mangostin’s long-term administration lessened the diabetic mice’s fasting-blood glucose without nephro- and hepato-toxicity, and PPARγ, AMPK, α-glucosidase, and α-amylase were the significant targets for its stimulatory binding. It exerted its hypoglycemic activity by boosting the uptake of glucose and lessening carbohydrate digestion through α-amylase/α-glucosidase inhibition with insulin sensitization [41]. γ-mangostin was reported to be a selective and potent SIRT2 inhibitor which displayed a potent antiproliferation capacity and increased the α-tubulin acetylation in MCF-7 and MDA-MD-231 cancer cells as well as inducing neurite outgrowth in N2a cells [42]. The docking study by Akawa et al. established the hSIRT2 (sirtuin-2) inhibition potential of α-, β-, and γ-mangostins, revealing their possible future development and design as sirtuin inhibitors for managing Alzheimer’s [43]. In addition, γ-mangostin attenuated Aβ (amyloid-β) 42 oligomers that caused OS (oxidative stress) and inflammation, thereby protecting the neurons from toxic injury and supporting its protective effectiveness in AD (Alzheimer’s disease) [44].

Additionally, *G. mangostana*’s benzophenones demonstrated cytotoxic activity and inhibitory potential for α-amylase, fatty acid synthase (FAS), and AGEs (Advanced glycation end products) formation [45,46].

**Figure 1:**
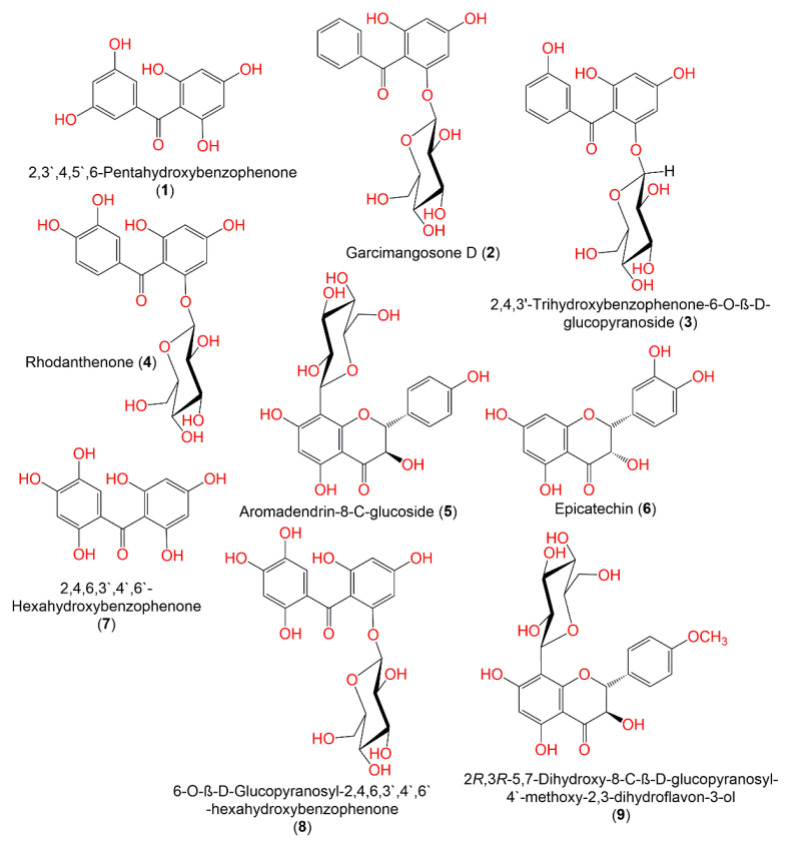
Structures of compounds **1**–**9**.

**Figure 2:**
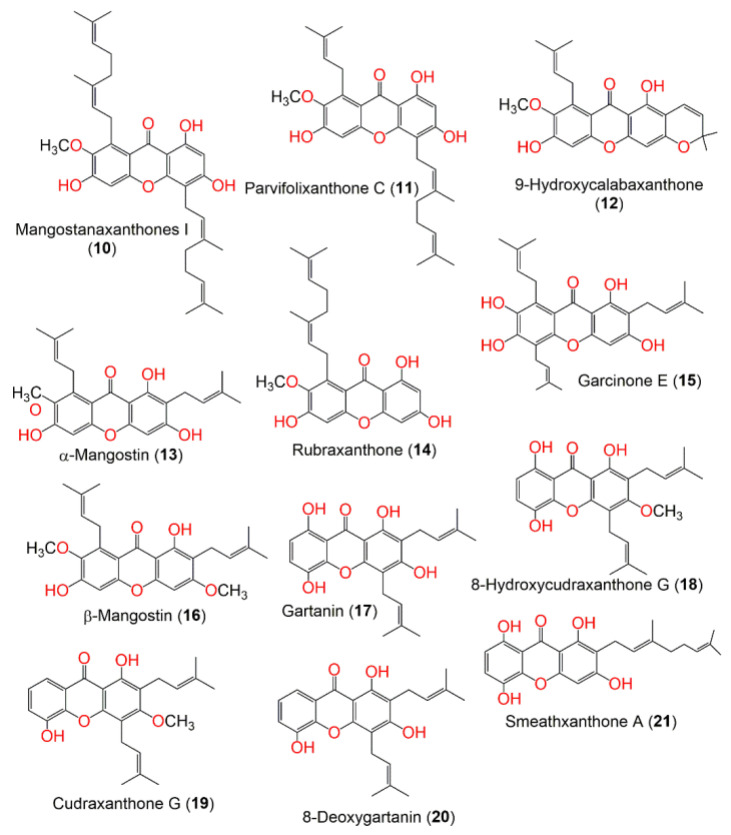
Structures of compounds **10**–**21**.

**Figure 3:**
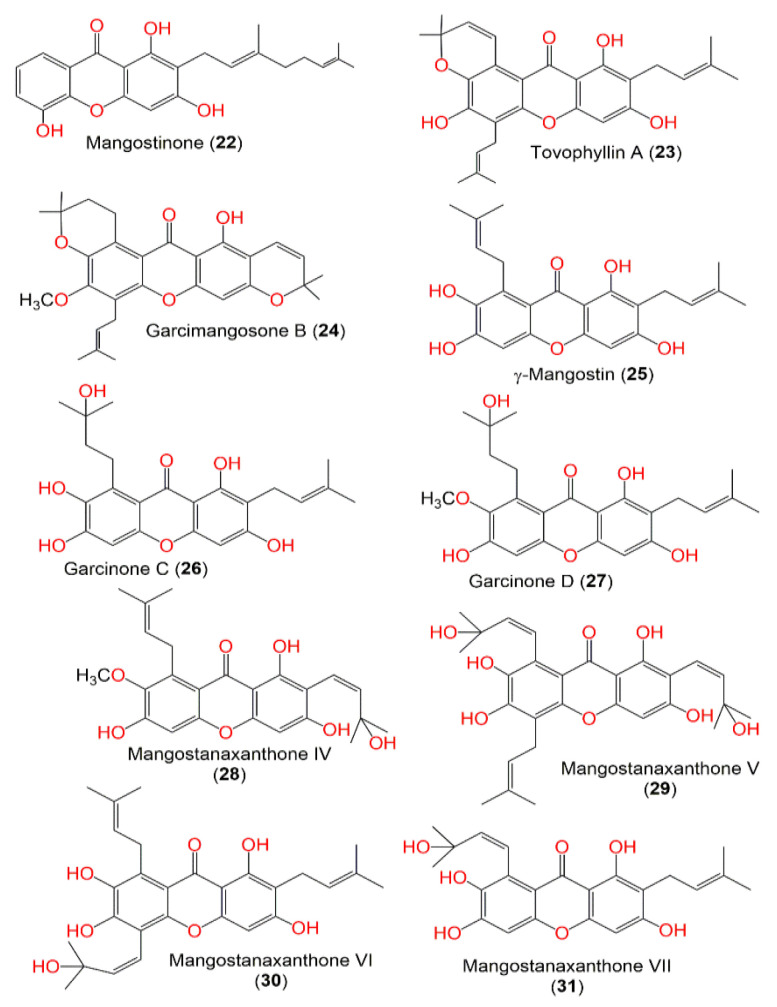
Structures of compounds **22**–**31**.

**Figure 4:**
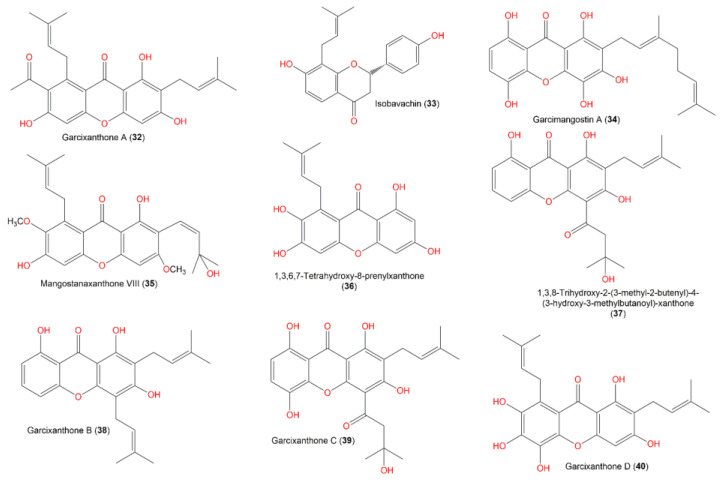
Structures of compounds **32**–**40**.

In continuation of the effort to discover the bioactivities of *G. mangostana* phytoconstituents, some metabolites reported from this plant by our group were screened in the current work to specify the potential metabolites that can function as alpha-amylase inhibitors using in vitro assays, and computational studies [24,25,26,27,28,29,30,31,32] (Figure 1, Figure 2, Figure 3 and Figure 4). The computational investigations used included grid generation followed by the docking of ligands as a second stage of screening with different scoring methods. Subsequently, the free energy of binding between the ligand and protein was calculated using MM-GBSA. After that, the metabolite with the highest potential was computationally verified by MD (molecular dynamics) simulations. Lastly, the in silico results were confirmed by carrying out in vitro alpha-amylase inhibitory assays for the top 10 hits.

## 2. Materials and Methods

### 2.1. Preparation of Protein

To carry out the docking assessment, the crystal structure of the alpha-amylase (PDB-ID: 4GQR) was imported from the protein_data_bank (Protein Data Bank; available online). Before docking, protein preparation was carried out through the Schrödinger suite protein preparation wizard tool [32]. The hydrogen and heavy atoms were subjected to optimization through restrained minimization. Missing hydrogen atoms were included, and the correct charges were allocated using the OPSL4 force field. Molecules of water beyond 5 Å from HETgroups were removed.

### 2.2. Ligand Preparation

Lig Prep was used to convert the 40 compounds from 2D to 3D structures [47]. Water molecules beyond 3 Å from HET groups were deleted, H-bonds were optimized using PROPKA at pH 7.0, and the OPLS4 force field was utilized for restrained minimization. Metals HET states and cofactors were set at 7.0 ± 2.0 pH.

### 2.3. Receptor Grid Generation and Molecular Docking

Grid generation and ligand docking were performed with the use of Glide [48]. The grid box was defined by selecting the co-crystallized inhibitor of 4GQR, and the binding region was defined using Glide’s Receptor-Grid-Generation tool. For docking the 31 prepared ligands, the grid generated by Glide was used in the Glide software. The selected protocol was XP (extra-precision). The default 1.0 radii scaling factor (vdW) and 0.25 potential charge cut-off were set. The co-crystallized ligand, myricetin, and acarbose were redocked using the extra precision (XP) protocol. The remaining settings were kept at default.

MM-GBSA (molecular mechanics-generalized born surface area) was calculated utilizing Prime for re-scoring the ligands’ docked poses [48]. The following equation was utilized to calculate the binding free energy (Δ*G_bind_*):

Δ*G_bind_* = *E_Complex_* (*minimized*) − *E_ligand_* (*minimized*) − *E_protein_* (*minimized*)

where the protein-ligand complex is (Complex), the free protein is (*E_protein_*), and the free ligands are (*E_ligand_*).

### 2.4. Molecular Dynamic Simulation (MDS)

The molecular dynamic simulations were performed utilizing the Schrodinger package Desmond software [49]. The chosen protein-ligand complexes were immersed into the simple point charge water box, which extended 10 Å beyond the atoms of the complex. System neutralization was achieved by adding Cl and Na counter ions. The simulation was performed at a 300 K temperature, with 1.01325 bar pressure. The force field was set at OPLS4 over 100 ns/trajectory, with the number of atoms, pressure, and temperature maintained constant (NPT ensemble). Figures and plots were sketched with the Maestro Desmond simulation interaction diagram tool.

### 2.5. Prediction of ADMET Properties

Maestro Schrodinger QikProp module [50] was used for obtaining the ADME properties of the compounds. Absorption, metabolism, distribution, elimination, and many other processes are crucial to the drug development process as they determine the compounds’ drug-likeness.

### 2.6. Alpha-Amylase Inhibition Assay

To verify the docking findings, compounds **3**–**6**, **8**–**10**, **13**, **15**, **17**, **23**, **28**–**31**, **35**, **37**, **39**, and **40** that were formerly purified by our group from *G. mangostana* pericarps were assessed for their alpha-amylase inhibition potential using an EnzChek^®^Ultra-Amylase-Assay Kit (Thermo-Fisher-Scientific Inc., Waltham, MA, USA), as formerly stated [24,25,26,27,28,29,30,31,32].

## 3. Results and Discussion

Seeking novel natural α-amylase inhibitors is of ultimate interest to the pharmaceutical industry. These metabolites could serve as potential components of pharmaceuticals or nutraceuticals, which could lessen diabetes-linked health burdens and improve the economic advantage of the industry. In this research, an in silico assessment of *G. mangostana* metabolites as α-amylase inhibitors were carried out.

### 3.1. In Silico ADME Properties of Selected Ligands

The examined metabolites of *G. mangostana* were analyzed for their ADME properties (absorption, distribution, metabolism, excretion) using QikProp and are detailed in Table 1. Analyzing the ADME predicts the drug-likeness, physicochemical properties, and biological functions of the compounds, which helps in the evaluation of the usefulness of the drug. Descriptors or factors such as molecular weight, drug-likeness, dipole moment, hydrogen bond donors and acceptors, aqueous solubility, brain/blood partition coefficient, binding to human serum albumin, central nervous system activity, and human oral absorption were predicted for the selected compounds. Some of the obtained values were within the recommended range, while others (highlighted) had some issues, such as poor oral absorption, concern with the blocking of the HERG K+ channels, or a few metabolic reactions, except for some values (Table 1). However, the analysis of the ADME descriptors for some compounds that have a high binding docking score like **8** needs more derivatization in the molecular structure to enhance its descriptors, such as donor hydrogen bonds, blockage of HERG K+ channel, a few possible reactions, and percentage of human oral absorption. It is clear that compound **8** has poor human oral absorption since most of the parameters that determined its absorption are outliers based on Lipinski’s rule of five [51].

### 3.2. Protein and Ligand Preparation

The 2D structures’ conversion to 3D, and ionization and tautomerization via LigPrep resulted in minimized 3-dimensional molecular structures, and the minimized 3D structures were docked with the alpha-amylase crystal structure PDB-ID: 4GQR. The alpha-amylase was also prepared via the protein preparation wizard tool, where H bonds were optimized and geometry was minimized. To confirm the assignment of the correct formal charges and force field treatment, missing hydrogens and the correct ionization state were added.

### 3.3. Molecular Docking Studies

After selecting the grid box in the prepared alpha-amylase via the Receptor-Grid-Generation tool from Glide in Maestro, the resultant 3D structures were docked into the co-crystallized binding area of the alpha-amylase inhibitor. The docked ligands were arranged and scored based on the most negative docking XP G.score, as shown in Table 2. These scores represented the ligands bound to the alpha-amylase with the best conformations and relative binding affinities. Among the tested compounds, **4**, 5, **6**, **8**, and **40** had high XP G.scores and Glide G.scores of −11.048, −11.855, −11.135, −12.425, and −10.989 Kcal/mol, respectively. Compound **8** possessed the highest XP and Glide docking score (−12.425 Kcal/mol) compared to the reference compounds myricetin and acarbose, which had XP and Glide docking scores of −12.319 and 11.201 Kcal/mol, respectively.

**Figure 5:**
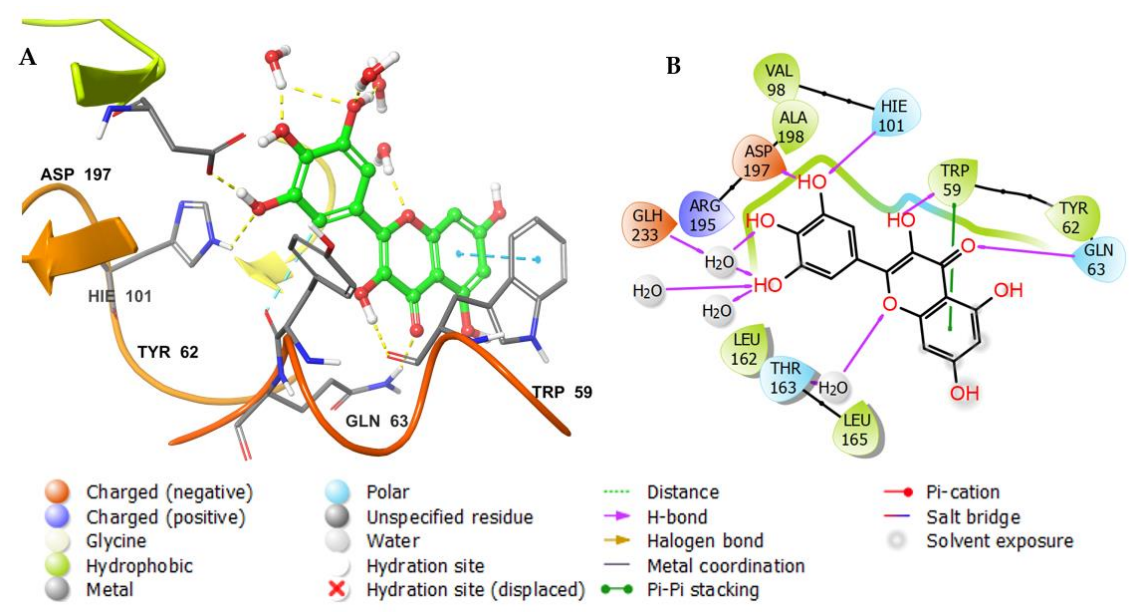
(**A**) Putative binding mode of the native inhibitor (myricetin) in the binding site of alpha-amylase (PDB: 4GQR). The native inhibitor is displayed as green sticks. The amino acid residues of the binding site are represented as thin tubes with color elements; pi-pi stacking and H-bonds are represented in cyan and yellow dotted lines, respectively. (**B**) 2D depiction of the ligand–protein interactions.

**Figure 6:**
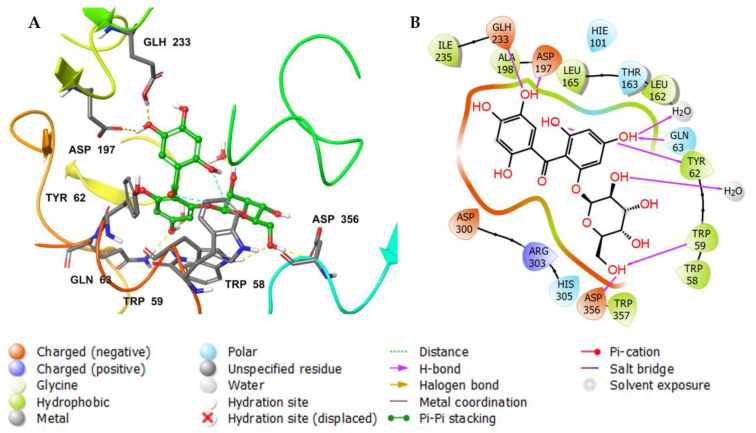
(**A**) Putative binding mode of **8** in the binding site of alpha-amylase (PDB: 4GQR). The inhibitor is displayed as green sticks. The amino acid residues of the binding site are represented as thin tubes with color elements; pi-pi stacking and H-bonds are represented in cyan and yellow dotted lines, respectively. (**B**) 2D depiction of the ligand–protein interactions.

**Figure 7:**
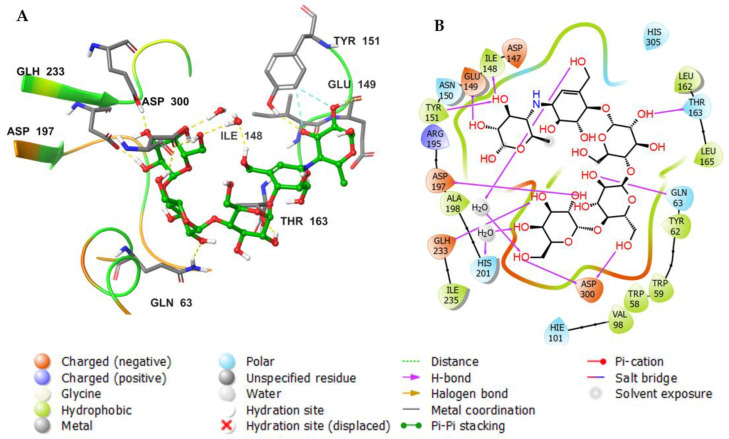
(**A**) Putative binding mode of acarbose in the binding site of alpha-amylase (PDB: 4GQR). The inhibitor is displayed as green sticks. The amino acid residues of the binding site are represented as thin tubes with color elements; pi-pi stacking and H-bonds are represented in cyan and yellow dotted lines, respectively. (**B**) 2D depiction of the ligand–protein interactions.

The docking of a molecule into the binding site of its target protein is a useful way to identify the correct binding pose among any predicted poses of a compound. The docking of the native inhibitor, myricetin (Figure 5), **8** (Figure 6), and acarbose (Figure 7), in the alpha-amylase was performed. For docking validation, the selected native inhibitor (myricetin) was prepared and redocked alongside the tested compounds, and the docking poses were examined by comparing the docked pose with the original pose of myricetin in the crystal structure. The RMSD was 0.2007. The native inhibitor, myricetin, through its hydroxyl groups, formed hydrogen bonds with the amino acid residues Tyr 62, Trp 59, His 101, Gln 63, and Asp 197, as well as pi-pi stacking with the residues Tyr 62 (Figure 5a,b). Compound **8** similarly interacted through its alcoholic and phenolic hydroxyl groups with Trp 58, Trp 59, Gln 62, Asp 179, Glh 233, and Asp 356 (Figure 6a,b). Both compounds form similar interactions with the catalytic residues and give the same effect, which is the hydrolysis of α-1−4 glycosidic bonds in starch. However, the difference in the binding with other residues could explain the differences in the docking scores. Acarbose (Figure 7a,b) binds to the catalytic region of the human alpha-amylase and forms hydrogen bonds between its alcoholic groups with Gln 63, Ile 148, Glu 149, Tyr 151, Asp 197, Glh 233, and Asp 300.

The docking of the tested compounds in the active site is a robotic method aimed at getting the right binding pose among several predicted poses for the compounds. However, we repeated the docking of the tested compounds with different docking protocols and different calculation methods (docking score, Glide G.score, XP G.score, and Glide emodel) in order to have an accurate rank based on the affinities of the tested compounds with the protein. Moreover, the MM-GBSA calculation was performed to predict and determine the binding energies: the values with more negative scores represent a stronger binding (Table 2).

### 3.4. Molecular Dynamics (MDs)

Though efforts have been undertaken to improve the docking calculations and predictions, we still end up with a motionless perspective of the compound’s binding pose in the protein’s binding site. The best way to compute and evaluate the atom movements during a specific period of time is by using molecular dynamic simulation by incorporating the classic equation of motion established by Newton [52]. The dynamic behavior of the protein–ligand complex stability in the system was stimulated via the MD simulation, and, for these purposes, the inhibitor with the best docking scores (**8**), the native inhibitor (myricetin), and acarbose were subjected to MD studies using the Desmond software (Supplementary Materials). The MD simulations were run with a simulation time of 100 ns, and the structures of the complexes were optimized at pH 7.0 ± 2.0. The stability of the complexes was predicted by analyzing the interaction map and the root mean square deviation plot of the protein and ligand.

**Figure 8:**
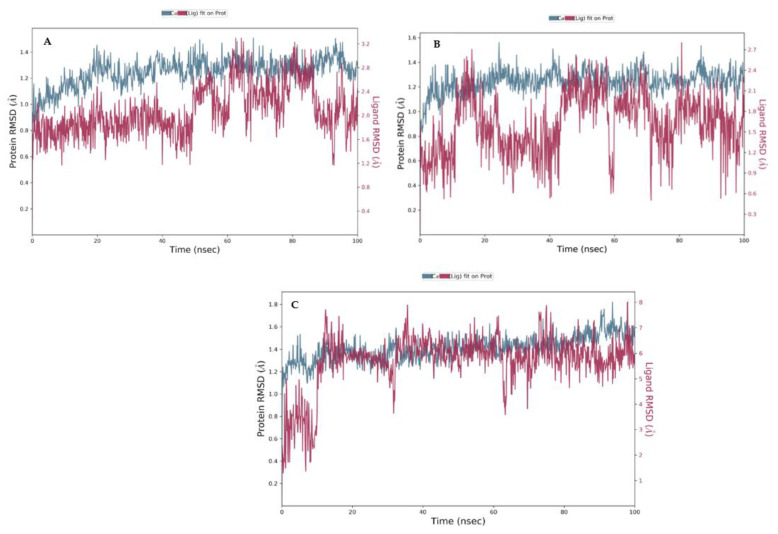
RMSD analyses for (**A**) native inhibitor (myricetin); (**B**) compound **8**; (**C**) acarbose with the alpha-amylase (PDB 4GQR) of MD simulation trajectory.

The RMSD plot of the human alpha-amylase with myricetin (Figure 8a), the plot of the alpha-amylase with **8** (Figure 8b), and acarbose (Figure 8c) indicates the stability of the complexes during the 100 ns simulation with regard to the reference time of 0 ns. The plot reveals the RMSD of the alpha-amylase on the left y-axis, while the y-axis on the right shows the ligand RMSD profile aligned on the protein backbone. There was a slight fluctuation in the native inhibitor’s plot (Figure 8a) and the tested compound (Figure 8b) at the time of the simulation, but they were within the acceptable range of 1–3 Å; therefore, it can be considered non-significant.

**Figure 9:**
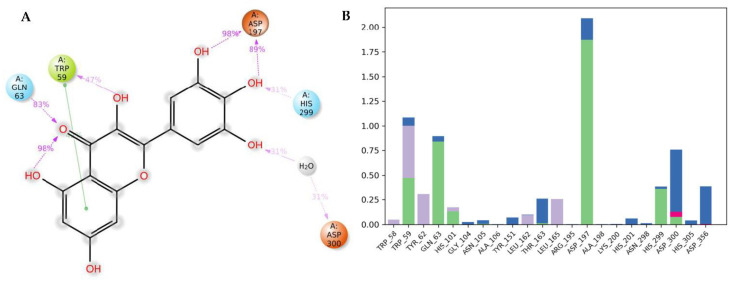
Analysis of molecular interactions and types of contacts with alpha-amylase after MD simulation. (**A**) Detailed schematic interaction of myricetin with the binding site residues of alpha-amylase in color scheme, where orange represents the charged, blue represents the polar, and light green represents the hydrophobic residues (PDB ID: 4GQR). (**B**) Normalized stacked bar chart of alpha-amylase binding site residues interacting, with the native inhibitor showing the main types of bonding interaction: hydrogen bonds (green), hydrophobic (violet), ionic (red), and water bridges (blue).

Figure 9a describes the detailed scheme of myricetin atom interactions with the amino acid residues. Interactions that lasted for more than 30.0% of the simulation time in the chosen frame (0.00 through 100.00 ns) are represented. The docked poses were kept throughout the simulation time of 100 ns. Molecular interactions in the form of H bonds between residues Trp 59 (59%), Gln 63 (83%), Asp 197 (89 and 89%), His 300 (31%), and Asp 300 (31% through water bridge) are displayed; myricetin is shown to have a hydrophobic interaction in the form of π-π interaction with Tyr 62. Figure 9b shows interactions that are classified into three main types and represented as stacked bars: hydrogen bonds (green), ionic (red), water bridges (blue), and hydrophobic (violet). The stacked bar charts were normalized over the course of the trajectory. A hydrogen bond with the residue Asp 197 was maintained for more than 100% of the trajectory time; this might be because of the various hydrogen bonds of the same subtype. Another hydrogen bond was formed with the residue Gln63 and maintained for more than 50% of the trajectory time. The same residues also formed water-bridged interactions. Multiple hydrophobic interactions were formed with different amino acid residues, including Trp 58, Trp 59, and Leu 162.

**Figure 10:**
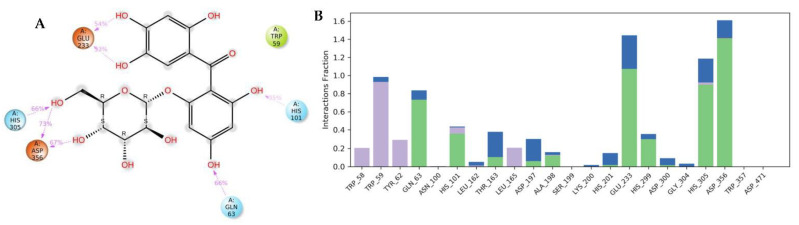
Analysis of molecular interactions and types of contacts with alpha-amylase after MD simulation. (**A**) Detailed schematic interaction of **8** with the binding site residues of alpha-amylase in color scheme, where orange represents the charged, blue represents the polar, and light green represents the hydrophobic residues (PDB ID:4GQR). (**B**) Normalized stacked bar chart of the alpha-amylase binding site residues interacting, with **8** showing the main types of bonding interaction: hydrogen bonds (green), hydrophobic (violet), and water bridges (blue).

Figure 10a shows a detailed scheme of the binding interaction of **8** with the protein residues that persist for at least 30% of the simulation time. Molecular interactions in the form of H bonds between residues Gln 63 (66%), His 101 (35%), Glu 233 (54% and 33%), His 305 (66%), and Asp 356 (73% and 67%) are displayed; hydrophobic bonds are shown with Trp 59. Figure 10b shows interactions that are classified into three main types and represented as stacked bars: hydrogen bonds (green), hydrophobic (violet), and water bridges (blue). The stacked bar charts were normalized over the course of the trajectory. The hydrogen bonds with the following residues showed varying degrees of maintenance throughout the trajectory time: Gln 63 (76%), His 101 (35%), Glu 233 (110%), His 299 (30%), His 305 (90%), Asp 356 (130%). The particularly high interaction percentage (>100%) might have resulted from the formation of multiple hydrogen bonds of the same subtype. Most of these residues were also formed by water-bridged interactions. Multiple hydrophobic interactions were formed with different residues, including Trp 58 (20%), Trp 59 (96%), Tyr 62 (24%), and Leu 165 (18%).

**Figure 11:**
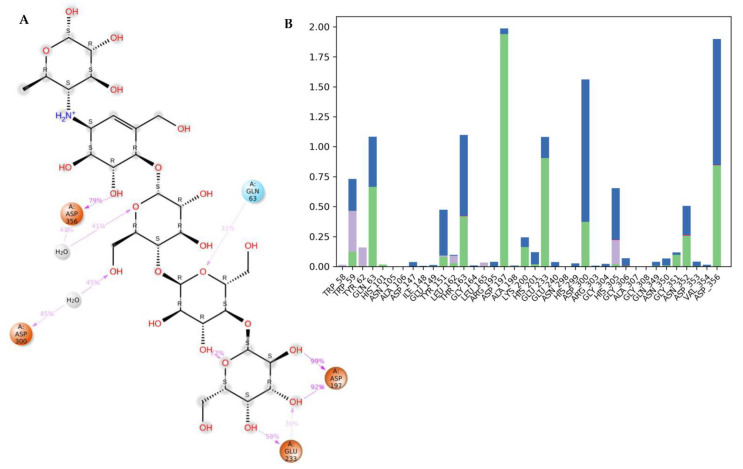
Analysis of molecular interactions and types of contacts with alpha-amylase after MD simulation. (**A**) Detailed schematic interaction of acarbose with the binding site residues of alpha-amylase in color scheme, where orange represents the charged, blue represents the polar, and light green represents the hydrophobic residues (PDB ID:4GQR). (**B**) Normalized stacked bar chart of alpha-amylase binding site residues interacting with acarbose showing the main types of bonding interaction: hydrogen bonds (green), hydrophobic (violet), and water bridges (blue).

Figure 11a shows a detailed scheme of the binding interaction of acarbose with the protein residues that persist for at least 30% of the simulation time. Molecular interactions in the form of H bonds between residues Gln 63 (31%), Asp 197 (99% and 92%), Gln 233 (30% and 59%), Asp 300 (54% through water bridges), and Asp 356 (79% and 41% through water bridge) are displayed. Figure 11b shows interactions that were classified into three main types and represented as stacked bars: hydrogen bonds (green), hydrophobic (violet), and water bridges (blue). The stacked bar charts were normalized over the course of the trajectory. The hydrogen bonds with the following residues showed varying degrees of maintenance throughout the trajectory time: Gln 63 (70%), Thr 163 (35%), Asp 197 (190%), Glu 233 (80%), Asp 300 (30%), and Asp 356 (75%). The particularly high interaction percentage (>100%) might be due to the formed hydrogen bonds with the same subtype. These residues also formed prominent water-bridged interactions: Gln 63 (30%), Tyr 151 (35%), Thr 163 (60%), Asp 300 (120%), His 305 (30%), and Asp 356 (100%).

### 3.5. In Vitro Alpha-Amylase Inhibitory Assay

The alpha-amylase inhibitory potential of 10 docked metabolites with the highest negative docking scores was assessed. The results revealed that all examined compounds revealed potent to weak alpha-amylase inhibitory potential, with IC_50_ ranging from 7.1–46.5 µM. It was noted that compounds **4**, **5**, **6**, **8**, and **40** had the highest XP G.score and Glide G.score, demonstrating the most powerful in vitro effectiveness (IC_50_s 7.1–11.1 µM) compared to acarbose (IC_50_ 6.7 µM) where 8 was the potent one with IC_50_ 7.1 µM (Table 2).

## 4. Conclusions

An in silico study helps in understanding how the metabolites may bind and exert their inhibitory potential. It is obvious that a combined in vitro and in silico approach is substantial for screening the active metabolites to detect the supposed molecular interaction affinity. Molecular docking is an in silico technique that is generally employed to foresee the orientation among the ligand and receptor. It is utilized in drug discovery because it is a time-saving and inexpensive technique. This presented investigation demonstrated the marked AAI potential of various mangosteen metabolites. Additionally, combined drug-likeness, physicochemical, pharmacokinetic, and molecular docking investigations, as well as MM-GBSA, were carried out on alpha-amylase to define new possible anti-diabetes therapeutic candidates. The docking results revealed H-bonds and other interactions relating to the binding energy’s significance and metabolite complexes’ stability, and different amino-acid residues in the enzyme active site that grant them AAIs. The current results revealed that. from the screened metabolites, compound **8** was a significant alpha-amylase inhibitor in the in vitro assay as well as in the molecular docking studies, as it had better binding sites and interactions with this enzyme. However, further in vivo and mechanistic investigations of this compound are warranted to validate its antidiabetic potential in the prevention or treatment of diabetes. The present findings added additional in vitro and in vivo evidence of the potential of *G. mangostana* and its phenolic metabolites in reducing postprandial glycemia.

## Figures and Tables

**Table 1:** In silico predicted ADME properties of the tested metabolites.

Molecule	# Stars	# rtvFG	CNS	mol_MW	SASA	donorHB	accptHB	QPlogPo/w	QPlogHERG	QPPCaco	QPlogBB	# Metab	QPlogKhsa	PercentHumanOralAbsorption
Recommended range	(0.0–5.0)	(0–2)	(−2 inactive) (+2 active)	(130–725)	(300–1000)	(0–6)	(2.0–20.0)	(−2–6.5)	concern below −5	<25 poor, >500 great	(−3–1.2)	(1–8)	(−1.5–1.5)	(<25% poor; >80% high)
2,3′,4,5′,6-Pentahydroxybenzophenone (**1**)	0	0	−2	262.218	470.594	3	4	0.554	−4.527	21.754	−2.322	5	−0.373	54.131
Garcimangosone D (**2**)	0	1	−2	392.362	602.925	5	12	−0.474	−4.889	20.469	−2.663	6	−0.814	34.68
2,4,3’-Trihydroxy benzophenone-6-O-β-D-glucopyranoside (**3**)	1	1	−2	408.361	613.179	6	13	−1.068	−4.798	7.724	−3.154	7	−0.905	23.626
Rhodanthenone (**4**)	4	1	−2	424.36	616.186	7	13	−1.664	−4.537	2.94	−3.583	8	−0.982	0
Aromadendrin-8-C-glucoside (**5**)	5	0	−2	450.398	681.978	7	14	−1.532	−5.347	2.688	−3.702	11	−0.852	0
Epicatechin (**6**)	0	0	−2	290.08	519.049	4	6	0.098	−4.869	21.252	−2.3	6	−0.426	51.276
2,4,6,3′,4′,6′-hexahydroxybenzophenone (**7**)	0	0	−2	278.218	474.191	3	4	0.561	−4.376	13.745	−2.542	6	−0.35	37.642
6-O-β-D-Glucopyranosyl-2,4,6,3′,4′,6′-hexahydroxybenzophenone (**8**)	5	1	−2	440.36	663.47	7	13	−1.65	−5.119	1.514	−4.259	9	−1.028	0
2R,3R-5,7-Dihydroxy-8-C-β-D-glucopyranosyl-4-methoxy-2,3-dihydroflavon-3-ol (**9**)	2	0	−2	464.425	708.735	6	14	−0.782	−5.418	8.732	−3.231	11	−0.769	13.292
Mangostanaxanthone I (**10**)	4	0	−2	546.702	951.529	2	5	7.657	−6.17	366.708	−2.059	16	1.967	91.76
Parvifolixanthone C (**11**)	2	0	−2	478.584	815.402	2	5	5.954	−5.517	369.062	−1.714	13	1.369	94.798
9-Hydroxycalabaxanthone (**12**)	0	0	0	408.45	671.549	1	5	4.793	−5.212	1503.476	−0.575	6	0.896	100
α-Mangostin (**13**)	1	0	−2	410.466	693.506	2	5	4.655	−5.125	882.919	−1.013	10	0.813	100
Rubraxanthone (**14**)	1	0	−2	410.466	676.001	2	5	4.469	−5.042	596.126	−1.209	10	0.733	100
Garcinone E (**15**)	2	0	−2	464.557	796.788	3	5	5.513	−5.427	485.861	−1.511	13	1.178	94.349
β-Mangostin (**16**)	2	0	−1	424.493	748.032	1	5	5.511	−5.493	1511.156	−0.829	10	1.103	100
Gartanin (**17**)	1	0	−2	396.439	687.965	2	4	4.512	−5.282	304.523	−1.524	10	0.9	100
8-Hydroxycudraxanthone G (**18**)	1	0	−2	410.466	696.392	1	4	5.004	−5.163	369.475	−1.415	10	1.095	89.241
Cudraxanthone G (**19**)	2	0	−1	394.466	706.416	1	4	5.273	−5.489	1075.829	−0.897	9	1.085	100
8-Deoxygartanin (**20**)	1	0	−1	380.44	667.452	2	4	4.546	−5.262	782.505	−1	9	0.813	100
Smeathxanthone A (**21**)	1	0	−2	396.439	710.514	2	4	4.445	−5.747	146.974	−2.007	10	0.905	91.759
Mangostinone (**22**)	1	0	−2	380.44	697.748	2	4	4.513	−5.815	352.029	−1.507	9	0.841	100
Tovophyllin A (**23**)	2	0	−2	462.541	784.186	2	5	5.712	−5.601	755.786	−1.111	9	1.323	100
Garcimangosone B (**24**)	2	0	0	476.568	784.948	0	5	6.434	−5.468	2337.985	−0.369	6	1.526	100
γ-Mangostin (**25**)	1	0	−2	396.439	675.17	3	5	3.803	−5.104	289.754	−1.511	10	0.595	93.281
Garcinone C (**26**)	1	0	−2	414.454	700.302	4	5	3.29	−5.268	123.883	−2.072	9	0.397	83.673
GarcinoneD (**27**)	1	0	−2	428.481	671.867	3	5.25	3.932	−4.683	433.902	−1.372	9	0.524	100
Mangostanaxanthone IV (**28**)	0	0	−2	426.465	691.651	3	5	4.098	−4.918	588.906	−1.233	8	0.594	100
Mangostanaxanthone V (**29**)	1	0	−2	496.556	788.467	5	6	4.363	−4.975	185.916	−2.009	9	0.724	80.147
Mangostanaxanthone VI (**30**)	2	0	−2	480.557	798.713	4	5	4.955	−5.285	295.03	−1.792	11	0.954	100
Mangostanaxanthone VI (**30**)	1	0	−2	480.557	794.228	4	5	4.842	−5.262	275.224	−1.827	11	0.918	100
Mangostanaxanthone VII (**31**)	0	0	−2	412.438	689.283	4	5	3.301	−5.171	184.105	−1.805	8	0.396	86.814
Garcixanthone A (**32**)	1	0	−2	422.477	716.018	1	5	4.725	−5.209	396.428	−1.407	9	0.942	100
Isobavachin (**33**)	0	0	−1	324.376	562.774	2	4	3.082	−4.496	389.48	−0.954	7	0.412	91.356
Garcimangostin A (**34**)	1	0	−2	412.438	710.47	3	4	3.756	−5.504	69.776	−2.389	11	0.667	81.934
Mangostanaxanthone VIII (**35**)	0	0	−1	440.492	724.392	2	5	4.855	−5.094	1113.098	−0.982	8	0.827	100
1,3,6,7-tetrahydroxy-8-prenylxanthone (**36**)	0	0	−2	328.321	550.081	3	5	2.08	−4.565	147.3	−1.514	7	0.055	77.93
1,3,8-Trihydroxy-2-(3-methyl-2-butenyl)-4-(3-hydroxy-3-methylbutanoyl)-xanthone (**37**)	0	0	−2	412.438	677.829	0	4	4.66	−5.009	139.018	−1.856	8	0.962	92.589
Garcixanthone B (**38**)	1	0	−2	380.44	657.216	1	3	5.031	−4.999	605.344	−1.083	9	1.088	93.235
Garcixanthone C (**39**)	1	0	−2	428.438	704.892	1	4	4.215	−5.258	75.781	−2.296	9	0.871	85.268
GarcixanthoneD (**40**)	1	0	−2	412.438	706.392	4	5.25	3.144	−5.245	86.596	−2.214	11	0.436	80.031

# Stars: number of property or descriptor values that fall outside the 95% range of similar values for known drugs. More stars reveal that a molecule is less drug-like than molecules with fewer stars; SASA: total solvent-accessible surface area in square angstroms utilizing a probe with a 1.4 Å radius; Dipole: computed dipole moment of the molecule; Acceptor H-bond: estimated number of hydrogen bonds that the solute would accept from water molecules in an aqueous solution; Donor H-bond: H-bonds estimated number that the solute would donate to H_2_O molecules in an aqueous solution; QPlogS: predicted aqueous solubility, log S; QPlogPo/w: predicted octanol/water partition coefficient; QPlogkhsa: prediction of binding to human serum albumin; QplogBB: predicted brain/blood partition coefficient; No. of Metabolites: number of likely metabolic reactions; % Human Oral Absorption: predicted human oral absorption on 0 to 100% scale; CNS: predicted central nervous system activity on a –2 (inactive) to +2 (active) scale; QPlogHERG: predicted IC_50_ value for blockage of HERG K^+^ channels; #rtvFG: reactive functional groups number; the specific groups are listed in the jobname.out file. The presence of these groups can lead to false positives in HTS assays and to reactivity, decomposition, or toxicity problems in vivo.

**Table 2:** Results of in silico screening against alpha-amylase (PDB: 4GQR) and in vitro inhibition assay.

Compound No.	XP G.Score	Glide G.Score	Docking Score (Kcal/mol)	Glide Emodel	Prime Energy	MMGBSA dG Bind	IC_50_ (µM)
6-O-β-D-Glucopyranosyl-2,4,6,3′,4′,6′-hexahydroxybenzophenone (**8**)	−12.425	−12.425	−12.227	−73.234	−21,238.5	−24.48	7.1
Myricetin	−12.319	−12.319	−12.319	−68.813	−21,398.5	−51.23	-
Acarbose	−12.201	−12.201	−12.201	−75.139	−20,979.6	−15.9	6.7
Aromadendrin-8-C-glucoside (**5**)	−11.855	−11.855	−11.823	−58.225	−21,216.7	−36.82	8.3
Epicatechin (**6**)	−11.135	−11.135	−11.108	−61.064	−21,253.4	−43.73	8.9
Rhodanthenone (**4**)	−11.048	−11.048	−10.887	−65.449	−21,207.1	−44.87	10.4
Garcixanthone D (**40**)	−10.989	−10.989	−10.764	−53.919	−21,260.7	−41.35	11.1
Mangostanaxanthone V (**29**)	−10.254	−10.254	−10.088	−70.702	−21,346.8	−38.65	11.9
2R,3R-5,7-Dihydroxy-8-C-β-D-glucopyranosyl-4-methoxy-2,3-dihydroflavon-3-ol (**9**)	−10.107	−10.107	−10.094	−52.556	−21,192.9	−26.05	12.8
1,3,8-Trihydroxy-2-(3-methyl-2-butenyl)-4-(3-hydroxy-3-methylbutanoyl)-xanthone (**37**)	−9.99	−9.99	−8.574	−59.986	−21,411.4	−33.11	14.2
2,4,3’-Trihydroxy benzophenone-6-O-β-D-glucopyranoside (**3**)	−9.77	−9.77	−9.615	−64.384	−21,203.7	−19.85	15.8
Garcimangosone D (**2**)	−9.696	−9.696	−9.541	−56.996	−21,194.9	−23.33	-
γ-Mangostin (**25**)	−9.518	−9.518	−7.058	−54.095	−21,255.6	−2.33	-
Isobavachin (**33**)	−8.79	−8.79	−8.79	−59.656	−21,270.1	−35.87	-
2,4,6,3′,4′,6′-Hexahydroxybenzophenone (**7**)	−8.647	−8.647	−8.11	−43.182	−21,308.1	3.74	-
Garcixanthone C (**39**)	−8.617	−8.617	−6.406	−55.483	−21,348.2	−3.66	17.1
Garcimangostin A (**34**)	−8.367	−8.367	−8.248	−60.875	−21,243.7	−29.37	-
Garcinone E (**15**)	−8.245	−8.245	−8.114	−59.531	−21,285.8	−33.75	18.8
1,3,6,7-Tetrahydroxy-8-prenylxanthone (**36**)	−8.213	−8.213	−7.915	−50.339	−21,298.3	−33.28	-
Garcixanthone B (**38**)	−7.978	−7.978	−7.904	−58.072	−21,314.5	−25.91	-
Smeathxanthone A (**21**)	−7.976	−7.976	−7.864	−56.159	−21,321.2	−39.59	-
Mangostanaxanthone VII (**31**)	−7.871	−7.871	−6.501	−61.697	−21,260.9	−4.38	19.5
Mangostinone (**22**)	−7.843	−7.843	−6.507	−47.68	−21,267.2	−13.3	-
Tovophyllin A (**23**)	−7.646	−7.646	−6.164	−46.506	−21,311.1	−35.81	20.4
Gartanin (**17**)	−7.635	−7.635	−5.417	−52.868	−21,268	−11.2	21.2
Mangostanaxanthone VI (**30**)	−7.577	−7.577	−7.479	−61.529	−21,297.6	−24.81	21.9
2,3′,4,5′,6-Pentahydroxybenzophenone (**1**)	−7.502	−7.502	−7.094	−49.619	−21,321.2	−28.82	-
8-Deoxygartanin (**20**)	−7.293	−7.293	−4.769	−53.856	−21,244.6	−17.72	-
Cudraxanthone G (**19**)	−7.12	−7.12	−7.12	−55	−21,237.8	−44.68	-
Mangostanaxanthone I (**10**)	−7.107	−7.107	−6.924	−53.946	−21,323.1	−48.36	28.3
Garcinone C (**26**)	−7.098	−7.098	−6.962	−53.746	−21,319.8	−37.48	-
8-Hydroxycudraxanthone G (**18**)	−6.667	−6.667	−6.66	−49.088	−21,268.2	−41.81	-
β-Mangostin (**16**)	−6.62	−6.62	−5.284	−38.5	−21,270.8	−24.24	-
Parvifolixanthone C (**11**)	−6.566	−6.566	−6.381	−56.272	−21,326.6	−39.95	-
Mangostanaxanthone VIII (**35**)	−6.541	−6.541	−6.483	−45.301	−21,299.1	−30.71	40.6
α-Mangostin (**13**)	−6.323	−6.323	−5.038	−41.996	−21,302.6	−29.84	44.1
Mangostanaxanthone IV (**28**)	−6.293	−6.293	−4.907	−42.202	−21,323.6	−24.42	46.5
Garcinone D (**27**)	−5.823	−5.823	−4.457	−49.712	−21,335.2	−34.69	-
Garcixanthone A (**32**)	−5.082	−5.082	−3.764	−47.142	−21,352.5	−37.06	-
Garcimangosone B (**24**)	−4.978	−4.978	−4.976	−45.586	−21,289.8	−39.15	-

## Data Availability

The data presented in this study are available in article.

## Supplementary Materials

The following supporting information can be downloaded at: https://www.mdpi.com/article/10.3390/metabo12121229/s1. Details of molecular dynamics simulation for 6-O-β-D-glucopyranosyl-2,4,6,3′,4′,6′-hexahydroxybenzophenone (**8**), myricetin, and acarbose.
